# The Biological Significance of Evolution in Autoimmune Phenomena

**DOI:** 10.1155/2012/784315

**Published:** 2012-03-14

**Authors:** Carlos A. Cañas, Felipe Cañas

**Affiliations:** ^1^Rheumatology Unit, Fundación Valle del Lili, ICESI University, Avenida Simón Bolívar Cra. 98 No.18-49, Cali, Colombia; ^2^Fundación Valle del Lili, Medical School, Universidad del Valle, Cali, Colombia

## Abstract

It is an inherent part of living to be in constant modification, which are due to answers resulting from environmental changes. The different systems make adaptations based on natural selection. With respect to the immune system of mammals, these changes have a lot to do with the interactions that occur continuously with other living species, especially microorganisms. The immune system is primarily designed to defend from germs and this response triggers inflammatory reactions which must be regulated in order not to generate damage to healthy tissue. The regulatory processes were added over time to prevent such damage. Through evolution the species have stored “an immunological experience,” which provides information that is important for developing effective responses in the future. The human species, which is at a high level of evolutionary immunological accumulation, have multiple immune defense strategies which, in turn, are highly regulated. Imbalances in these can result in autoimmunity.

*“There is nothing permanent except change.”*

(Heraclitus)

## 1. Introduction

Life began on earth more than 3.5 billion years ago and evolution has allowed the development of myriads of species from very simple to highly complex ones. Initially, unicellular microorganisms without a nucleus (prokaryotes) similar to modern bacteria appeared and after that others with a nucleus similar to amoebas. These ancestral amoebas developed in groups and have been called “social amoebas.” These feed on bacteria in the soil and they aggregate when there is a serious lack of food to form a migrating group. A type of amoeba in these groups differentiated and facilitated the process of detoxification through immunological mechanisms and became what is called a sentinel (S-cell) [1]. The S-cell engulfs bacteria and sequesters toxins. This may be the origin of the immune system. Subsequently, these unicellular eukaryotes differentiated into diverse functions and developed forms of signaling and adhesion molecules which allowed them to aggregate. This could have been the beginning of evolution of multicellular organisms (metazoans) which later migrated from the sea [2]. Around 600 million years ago, terrestrial metazoans began to develop in conjunction with an important increase in the oxygen concentration in the atmosphere. The vertebrate animals with their remarkable diversification appeared 500 million years ago in a relatively short time period termed the “evolutionary big bang.” Since the beginning of life, the most important element of evolution has been the increasing ability of living things to accumulate information about these processes at different levels of memory.

Recently Dawkins [3], in his masterpiece, showed us that the different forms of memory were the most relevant foundation for evolution. Information required for handling the present so as to survive into the future is necessarily obtained from the past. In fact, he proposed four levels of information gathering, which he called the “four memories,” which would be the foundation of evolution. The “first memory” is DNA, the inherited database each species has, and which is the result of nonrandom evolution. It is the record of recurrent ancestral and cumulative details resulting from interaction with the environment which led to the primary characteristics of each species. The “second memory” is the adaptive immune system, which is cumulative information about dangerous microorganisms with which the individual interacts and through this interaction acquires the ability to defend itself from subsequent exposure with high efficiency. This process is present during the life of the individual. The “third memory” is the fact that we can think and it resides in the nervous system. By mechanisms that we do not yet fully understand, our brain records past experiences and works by a trial-and-error process that can be seen as yet another analogy to natural selection. In the human species, this level contains lists of faces, places, music, social customs, rules and words. The “fourth memory” is the collective memories inherited nongenetically from past generations and the culture. It also includes the information gathered through oral tradition and writing and, most recently, computer systems and Internet. This latter level of memory is necessary today for human survival.

Over time, the recorded information of repeated processes may be stored at a lower level of memory. Like wings or lungs that are necessary for survival, each adaptive change was developed as a secondary information-gathering system and later moved to the primary level. In fact, the mechanisms of evolution were also involved in the immune system development. The innate immunity which provides the early line of defense against microbes was developed by the perennial need for protection.

Based on these concepts, we are proposing a way to look at the autoimmune processes from the point of view of evolution with special attention to immune receptors which are crucial for sensing damage-causing agents and fine tuning the immune and inflammatory response that results. These receptors go through constant selection changes caused by the pressure of evolving pathogens. These pathogens stimulate the development of effective immune reactivity in order to maximize the destruction of the pathogens while avoiding an excessive immune and inflammatory response that could lead to consequences such as autoimmunity or septic shock [4].

**Figure 1:**
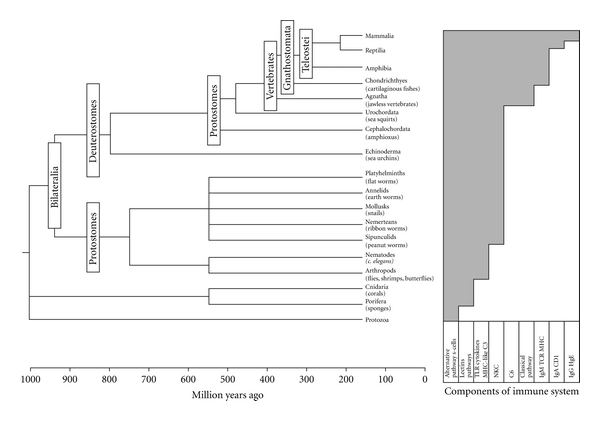
Phylogeny of animals and their immune system. Notice the form of accumulative evolution of the immune system from an innate to an adaptive system.

In Figure 1, the evolutionary pathways of animal species are traced and the way the immune system becomes more complex in cumulative strategies is outlined.

It is important to note that the majority of species alive today are not from species that still exist but from previous ones. That is, man did not descend from monkeys, but shares a common ancestor. Through the study of species existing today, it is possible to describe with some degree of precision what their ancestors were like and how they evolved. Many species have disappeared and we only know about them through their fossils [5].

## 2. Influence of the First Form of Evolutionary Memory on the Autoimmune Phenomena

Innate immunity is a system that does not create a new form of memory and should be included in the first memory of evolution. The innate immunity is natural, nonspecific, nonanticipatory, and does not generate an accumulation of information. This system contains cells that resemble phagocytes which have generic receptors that recognize conserved patterns of pathogens and lectine-like soluble proteins, and they are essential in arthropods, nematodes, sipunculids, mollusks, annelids, platyhelminths, echinoderms, cephalochordates, and urochordates. Adaptive immunity, which has a highly diverse repertoire of lymphocytes, was added in agnathas (vertebrates without jaws) and gnathostomes (vertebrates with jaws). In fact, in the most complex species like mammals, the immune system consists of innate and adaptive immunities which include T and B lymphocytes and the production of cytokines and antibodies.

### 2.1. The Pattern Recognition Receptors (PRRs)

 Human innate immunity shares similar cells, cellular structures, and molecules with invertebrates. The PRRs are of special interest. These include members of nucleotide oligomerization domain proteins containing leucine-rich repeats (NLRs), retinoic acid inducing gene (RIG)-like helicases (RLHs), and toll-like receptors (TLRs) [6]. TLRs deserve special attention and are one of the largest and best-studied PRRs. They are expressed on macrophages, dendritic cells, epithelial cells, and endothelial cells, where they provide rapid responses including the induction of proinflammatory cytokine secretion that recruits and activates additional immune responses. Such receptors were highly conserved during evolution and were first identified in *Drosophila melanogaster *[7]. The TLRs are necessary for defense from various microorganisms. For example, it has been demonstrated that mutant *Drosophila*, which carries loss-of-function mutations in the toll receptor, resulted in high susceptibility to fungi infection. The defective induction of an antifungal peptide provided the first evidence that *Drosophila *expresses a specific receptor responsible for sensing a fungi infection [8]. TLR has a leucine-rich extracellular domain interrupted by cysteine motifs and an intracytoplasmic domain similar to the interleukin-1 (IL-1) receptor all of which contain ITAMs domains that make it possible to follow the cascade of signaling events through phosphorylation of tyrosine residues [9]. This similarity between the TRL and the IL-1 receptor domains has the same phylogenetic origin in invertebrates and is highly conserved among them. Nowadays, this domain has been named TIR (Toll/1L-R). Unlike other human TLRs that are typically present on the surface of cells and recognize bacterial danger signals, a group of TLRs including TLR3, TLR7, and TLR9 localize cell endosomes and recognize viral danger signals (dsRNA, ssRNA, and hypomethylated dsDNA, resp.). This group of endosomal TLRs has been particularly implicated in the pathogenesis of autoimmune diseases. Human-derived RNAs and DNAs that are targets of autoimmune responses in systemic lupus erythematosus (SLE) and related conditions have been found to induce activation of these receptors [10]. Altered expression and function of these receptors have been linked to clinical manifestations of lupus-like autoimmunity in animal models [11, 12]. In the case of rheumatoid arthritis (RA), it has been postulated that after the activation of TLR by exogenous stimuli, these receptors recognize endogenous proteins. Potential endogenous TLR ligands are head shock protein (HSP)-60, HSP-70, gp96, high mobility group box 1 protein (HMGB-1), serum amyloid A, and low molecular weight hyaluronic acid. Therefore, they are capable of inducing a self-perpetuating inflammatory process which plays an important role in the pathogenesis of RA [13]. This is a form of autoimmunity related to innate immunity and it occurs when regulatory systems are scarce and ancestral mechanisms such as RNA interference (RNAi) are involved [14]. In the case of primitive animals that possessed only an innate defense system, such animals might have suffered occasional disregulation which resulted in reactions against their own bodies and thus, parallel mechanisms to prevent self-injury had to be developed [15].

### 2.2. Cytokines

 Proinflammatory cytokines and their receptors are present in early representatives of metazoans, such as cnidarians, and seem to be conserved in the entire animal kingdom. They are a family of secreted and regulatory molecules with a hormone-like activity and molecular mass ranging from 10 to 50 kDa. Cytokines are produced transiently and locally. Their mechanism of action is mainly paracrine or autocrine with the ability to induce a potent response in very small amounts. Cytokines interact with high-affinity cell surface receptors specific for each cytokine or cytokine group, which, when bound, leads to changes in the pattern of cellular RNA and protein synthesis [16]. They facilitate communication between cells, especially those of the haemopoietic and neuroendocrine systems. The evolution of the genes that encode the current spectrum of cytokines and receptor complexes involved multiple duplications from a smaller set of genes followed by the divergence of sequence and product function [17]. Recognition between distant cells is a phenomenon that is almost as old as metazoa itself dating back at least one billion years. A clear example of cell-to-cell recognition can be found in protozoa during sexual reproduction when recognition and signaling occurs between cells having a cell surface-associated set of “permissive” molecules which allow conjugation and exchange of genetic material between individual cells [18]. A marine ciliate protozoa (*Euplotes raikovi*) produces and releases specific pheromones into seawater which bind to receptors present on cells that are at the same point in the cell cycle and trigger molecular pathways which lead to a reciprocal search for permissive partners and to sexual reproduction [19, 20]. One of these pheromones was capable of binding to the *α* and *β* subunits of the IL-2 receptor on mammalian cells, and interleukin-2 (IL-2) was able to bind to its putative receptors on the ciliate protozoa cell surface. The implications of distant kinship suggested by these findings between IL-2 and pheromone families are supported by similarities in the structures of these molecules which suggest a conservation of this cell signaling system during evolution [21]. A similar cross reaction that confirms an ancestral relationship between ligands and receptors is seen in a cytokine-like factor with proinflamatory functions found in the blood of the starfish *Asterias forbesi*. This factor stimulates monocyte chemotaxis and macrophage activation in mammals [22, 23]. The mussel *Mytilus edulis *has been the subject of studies to determine whether the relationships between the immune and neuroendocrine systems, observed in vertebrates, may also be present in invertebrates. The effects of rIL-1 and tumor necrosis factor *α* (TNF-*α*) were studied in *Mytilus *hemocytes previously shown to produce and react to opioid peptides. These cells responded to these cytokines both *in vitro *and *in vivo*, in a manner similar to that of human granulocytes. In addition, the presence of immunoreactive IL-1 and TNF in *Mytilus *hemolymph was demonstrated using polyclonal antibodies against mammalian cytokines [24].

#### 2.2.1. IL-1

 The family of the IL-1 is made up of IL-1*α*, IL-1*β*, IL-18, which are proinflamatory cytokines, and the IL-1 receptor antagonist (IL-1ra) with pivotal roles in the regulation of acute inflammation. The inactive IL-1 precursors of mammals must be cleaved intracellularly by the IL-1 converting enzyme (ICE) to release the biologically active form [25]. The nonmammalian vertebrate IL-1 lacks the sequence coding for the ICE cleavage site and requires another mechanism to active the cytokine [26]. IL-1 activity occurs as a consequence of binding to its receptor complex (IL-1R) on the cell surface of target cells. The binding of IL-1 to its receptor triggers complex intracellular pathways that result in the activation of new genes or modification of proteins. As already mentioned, the intracellular domain of the IL-1 receptor is a “TIR domain” and is similar to the intracellular domain of TLR. TIR domains participate in host defense and inflammation and are present in mammals, insects, and plants [27]. An IL-1-like cytokine, which induces increased vascular permeability in rabbit skin, has been reported in ascidians. This effect was neutralized by a polyclonal antihuman IL-1 antiserum [28]. In humans, a different form of polymorphisms, SNPs, are implicated in the severity of a number of autoimmune diseases such as RA in which an adequate balance between IL-1 and IL-1ra is also required [29].

#### 2.2.2. TNF-*α*

 TNF*α*, mainly produced by monocytes/macrophages, regulates inflammation and cellular immune responses [30]. One of its functions is the modulation of the expression of IL-1, IL-6, and chemokines [31]. TNF-*α* requires a converting enzyme (a metalloproteinase), which generates a 17 kDa soluble mature peptide. The active form of TNF-*α* is a homotrimer that binds to two distinct receptors on the cell surface, TNFR1 and TNFR2, which elicit different cellular responses including cellular differentiation, proliferation, and apoptosis. Several proteins that interact with the cytoplasmic domains of these receptors have been identified and include the signaling cascades that lead to activation of NF-kB, c-Jun N-terminal kinase, and the apoptotic pathway. Teleost fish has TNF-*α* and TNF-*α* receptors and the human recombinant TNF-*α* produces biological effects such as macrophage respiratory burst activity, neutrophil migration, and lymphocyte proliferation [32]. A similar cross-reaction observed with IL-1 confirms an ancestral relationship with other species. Infliximab, a chimeric antibody in which the Fab portion has a mouse origin effectively blocks the human TNF*α* molecule and provides a clinical benefit for patients with active RA [33]. TNF-*α* and their receptors have been implicated in the pathogenesis of diverse autoimmune diseases with special interest in their polymorphisms [34]. A regulatory mechanism for reducing the inflammatory response in infections such as tuberculosis (TB) is presumably the development of polymorphism by natural selection. The −308 and −238 single nucleotide polymorphisms (SNP) of TNF-*α* may influence the presence of autoimmune diseases and TB. In fact, TNF −308G was both associated with TB and protective for autoimmunity, TNF −238A allele was protective for autoimmunity but represented a susceptibility factor for TB, and the haplotype −308A −238G was a protective factor against TB while, at the same time, it carried susceptibility for RA, SLE, and Sjögren's syndrome (SS) [35]. These results support the hypothesis that autoimmune diseases are a consequence of natural selection for enhanced TB resistance. Likewise, it is important to know that evolutionary mechanisms have been developed for the production of TNF-*α* regulatory mechanisms, and their disruption can lead to an increase in action and be associated with autoimmunity. Tristetraproline (TTP) is one of them. The TTP family of CCCH tandem zinc-finger proteins consists of three known members in mammals with a fourth member recently identified in frogs and fish [36]. TTP is now known to bind to the so-called class II AU-rich elements within the mRNAs that encode TNF-*α* and the granulocyte/macrophage colony-stimulating factor (GM-CSF). In both cases, this binding results in destabilization of the mRNA and decreased secretion of the protein. Recent evidence suggests that TTP can accomplish this accelerated mRNA degradation by first promoting removal of the polyadenylated tail from the mRNA (deadenylation) [37]. A TTP deficient mouse develops a deep inflammatory syndrome with erosive arthritis, autoimmunity, and myeloid hyperplasia [38]. In patients with RA, a low TTP/TNF-*α* gene expression ratio could indicate failure to produce adequate amounts of TTP in response to increased TNF-*α* production [39].

### 2.3. Complement System

Serine proteinases appeared early in evolution. They have even been found in bacteria [40] and evolved to supply several physiological needs in the immune system and others. A serine proteinase cascade which shows similarities to the blood clotting system and the complement system of vertebrates is involved. There is even a functional link between immunity and haemostasis, so coagulation factors activate immunological processes and various components of the complement also activate coagulation factors [41, 42]. Substrates of these protease cascades show evolutionary relationships. The complement system has more than 30 components. About one-fourth of them are serine proteases that are important for the activation or regulation of the system. The three branches of the complement in mammals are classical, lectin, and alternative pathways that converge in C3 protein and continue until the terminal phase with the C9 assembled to form the membrane attack complex (MAC). The complement system plays important roles not only in defense but also in normal tissue regeneration and development. The complement participates in the removal of immune complexes, aberrant and apoptotic cells, and cell debris and has important functions which, if they fail, are implicated in autoimmunity.

#### 2.3.1. Initiating Enzymes

 The classical pathway is triggered by antigen-antibody complexes and the proteases involved are C1r, C1s and C2. The lectin pathway is triggered by mannan-binding lectine and the proteases involved are mannan-binding-protein-associated serine proteases (MASP)-1, MASP-2, and MASP-3. The alternative pathway is triggered by pathogen and the proteases involved are factor D and factor B. The classical pathway is the newest phylogenetically and participates in the link between innate and adaptive immune systems. The deficiencies of C1q, C1r, C4, C2 are associated with SLE development by the failure to remove circulating immune complexes which may be deposited in blood vessel walls and tissues [43].

#### 2.3.2. C3

 The central component of the complement system is the C3 protein when the three pathways converge. It has been identified in jawless vertebrate and derives from a common ancestor, *α*-2-macroglobulin. This has been found in vertebrates, arthropods, and mollusks thus suggesting an early evolutionary origin and showing its importance as a defense molecule [44]. It contains an unstable internal thioether bond that, in nonactivated form, is buried in a hydrophobic pocket. When the protein is active, there is a cleavage of the thioether bond which allows the formation of a stable covalent bond with an adjacent substrate or water. C3 can be cleaved spontaneously in the alternative pathway or by other enzymes in the classical and lectin pathways. These processes must be regulated by proteases that promote proteolytic degradation of C3 (factor I and factor H in an alternative pathway), and their deficiency is associated with immune complex-mediated glomerulonephritis.

#### 2.3.3. MAC

 In the terminal complement component or lytic pathway, the C5, C6, C7, C8, and C9 proteins are present. The later configures the MAC which forms pores on the plasma membrane of the target cell, disturbs the membrane potential, and finally leads to cell lysis by a mechanism similar to perforin, which is the lytic protein of natural killer cells and cytotoxic lymphocytes [45]. Molecules homologous to mammalian C5 have been described in several species of teleost fish [46]. All these molecules share common structural motifs, that is, thrombospondin (TS), low-density lipoprotein receptor (LDL-R), and epidermal growth factor precursor (EGFP) domains. The cloning of a C6-like gene from the most primitive of present-day chordates, the amphioxus *Branchiostoma*, suggests an ancient origin of the C6/C7/C8/C9/perforin gene family. It seems reasonable for the duplication of an ancestral gene to have proceeded through these pathways. One pathway presumably led to the simple form of perforin while the second produced the ancestor of C6-C7 with its complex modular structure. Further duplication and loss of modules may have led to the creation of C8 and C9 molecules. The MAC present in teleost fish closely resembles the mammalian complex. CD59 is a regulatory factor of the terminal complement system. It blocks C9 binding and prevents the formation of MAC. Its deficiency is associated with hemolytic anemia.

### 2.4. Receptors for the Fc Region of IgG

(FcgR) provides a type of link between the humoral and cellular immune system. Inherited FcgR polymorphisms influence human phagocyte function. Single-aminoacid/SNP substitutions within the extracellular domains of FCgR alter the ability of the receptor to bind IgG and have been associated with the development of autoimmune and infectious diseases [47]. FCgRII (CD32) has two isoforms, FCgRIa and FCgRIIb, which are expressed on mononuclear phagocytes, neutrophils, and platelets. FCgRIIa has 2 codominantly expressed alleles, H131 and R131, which differ at aminoacid position 131 in the extracellular domain (histidine or arginine, resp.) and differ substantially in their ability to bind human IgG2 [48, 49]. H131 is the high-binding allele, R131 the low-binding allele and heterozygotes have an intermediate function [50]. FcgRIIa-H131 is essential for handling IgG2 immune complexes. These immune complexes are removed from circulation, primarily in the liver and spleen, by the mononuclear phagocyte system. Impaired removal of immune complexes is present in SLE which leads to an increase in the probability of tissue deposition of immune complexes, release of inflammatory mediators, influx of inflammatory cells, and damage to target-organs such as in the case of nephritis [51, 52]. FCgRIIb is the only FCgR inhibitor and regulates signalling which is crucial for the maintenance of B-cell tolerance and for the fine tuning of inflammatory and immune responses [53]. FCgRIIb-deficient mice are prone to inducible autoimmunity and, in some circumstances, develop spontaneous SLE [54]. In contrast, they are protected from both bacterial infection [55] and malaria [56]. In humans, several SNPs that modify the expressions or function of FCgRIIb have been described. A promoter polymorphism that may influence expression and be associated with SLE has been described [57]. An SNP in exon 5 of *FCGR2B *results in an isoleucine to threonine substitution within the transmembrane domain (1232T, rs1050501) which leads to the loss of the inhibitory function associated with the exclusion of FCgRIIb from lipid raft [58]. Homozygosis for this SNP is strongly associated with SLE [59, 60] and is found in 1% of Europeans, 5–7% of South-East Asian and Kenyans and in more than 10% of African-Americans. The high prevalence of this SNP in African and Asian populations may be due in part to the observation that it not only predisposes to SLE, but also protects children from malaria [61]. This is another example of how the effects of evolution based on natural selection can influence the genesis of autoimmune phenomena. Fc*γ*RIII (CD16) has two isoforms, Fc*γ*RIIIa and Fc*γ*RIIIb. Fc*γ*RIIIb has two codominantly expressed alleles: NA1 and NA2 with changes in the aminoacid sequences that may also alter the affinity to immune complexes. The first has a low binding to the immune complex and is more associated with autoimmune processes such as antineutrophil cytoplasmic antibody (ANCA)-positive systemic vasculitis [62]. In SS, there is a similar correlation with the presence of FCgRII [63] and FCgRIII [64] polymorphisms.

Therefore, we postulate that primitive animals with an innate immune system may have been attacked by their own system at different times during evolution and, consequently, developed regulatory mechanisms such as RNAi, TTP, IL-1ra, regulator complement cascade proteins, and FCgRIIb presented above. Another regulatory mechanism which is implicated in autoimmune phenomena when it fails is the one associated with the functions of suppressor T cells [65].

### 2.5. Class III Major Histocompatibility Complex (Class III MHC)

 This loci contains several genes that encode secreted proteins that play innate immune functions: components of the complement system (such as C2, C4 and factor B) and inflammation-related molecules (cytokines such as TNF-*α*, LTA, LTB) or HSP. Class-III has a completely different function classes-I and II (described below in the text), but is between the other two in the short arm of human chromosome 6.

## 3. Influence of the Second Form of Evolutionary Memory in Autoimmune Phenomena

Adaptive immunity is a form of second evolutionary memory and stores molecular information in microbes in order to have a quicker and more effective defense against them in future exposures through cytokines and specific antibodies. The most important mechanism that nature has used to obtain and retain this type of information has been the immunoglobulin superfamily gene system which provides information to create multiple receptors. The adaptive immune system, as defined by rearranging antigen receptor genes in the immunoglobulin superfamily and by the major histocompatibility complex, has only been found in the jawed vertebrates (gnathostomes). The mechanism of recombination-activating gene (RAG)-mediated rearrangement exists in all jawed vertebrates, but the organization and structure of immunoglobulin (Ig) genes, as they differ among fish and fish species, reveal their capability for rapid evolution. Recombination among these loci created hybrid genes, the strangest of which encodes variable (V) regions that function as part of secreted molecules and, as the result of an ancient translocation, are also grafted onto the T-cell receptor [66]. Other groups of proteins that belong to the immunoglobulin superfamily with a common ancestral origin are Fc*γ*R and immunoglobulin-like receptor (KIR) which have an important function in the infection inflammatory response and the alteration of which is associated with autoimmune-type responses.

### 3.1. Autoantibodies, Autoantigens

 The antibodies directed against their own structures (autoantibodies) have a primary role in autoimmunity being pathogenic in diseases caused by an attack on cell or tissue antigens (autoantigens), or in immune complex-mediated diseases. Several factors are implicated in this deviation from the primary role of the antibodies humeral immunity: (1) the formation of a repertoire of B cells that attack their own structures as they do not do adequate receptor editing or negative selection, (2) the recognition of autoantigens as foreign because they are similar to the structures of microorganisms (molecular mimicry), or (3) the possibility of not adequately removing immune complexes. The autoantigens may be from different sources.

#### 3.1.1. Protein Structures That Are Present in the Organs and Which Are “Visible” to the Immune System

 These molecules may have structural or functional roles and are usually common among mammals. Humans and mice share most of their genome. The passive immunization of mice with antibodies from human patients with autoimmune diseases can reproduce components of the diseases.

#### 3.1.2. Proteins That Are Hidden in the Tissues and by Factors Such as Trauma Begin to Be Recognized by the Immune System as Foreign

The system starts processes to eliminate them with innate mechanisms initially and after that with acquired immunity mechanisms. An example of this condition is the sympathetic ophthalmia (SO) where breaching of systemic ocular barriers compromises the relative immune privilege of the eye and causes sensitization to previously sequestered uveoretinal antigens [67]. A similar mechanism is observed in relapsing polychondritis which begins with cartilage trauma and is triggered by an immune response to other cartilaginous or noncartilaginous tissues [68].

#### 3.1.3. Even Though There Are Proteins That Are Not Present in the Human Structure, the Genetic Information Is Present

 These genes are ancestrally repressed and hidden. As a result of different causes, they start the protein synthesis and this “foreign” protein is attacked by the immune system. An example is the endogenous retrovirus (ERV) which belongs to the large family of retrotransposable elements of human genoma [69]. ERV may have originated from an exogenous retrovirus that integrated ancestrally into the genoma and became trapped owing to mutations of essential genes and is transmitted genetically in the classical Mendelian form [70]. ERV is strand RNA viruses with a mode of replication in which the RNA genome is transcribed into DNA by reverse transcriptase. ERV may be activated by radiation, bacteria, chemicals, or recombination with an exogenous retrovirus [71, 72] and starts the “autoantigen” protein synthesis that is the source for autoimmune processes implicated in the pathogenesis of SLE [73]. Another mechanism is the biological effect of a viral product. For example, certain components derived from endogenous retroviruses pl5E present in several species, including murine, feline, and human, induce the immune abnormalities observed in SLE lymphocytes [74].

#### 3.1.4. Ancestral Proteins Become Visible to the Immune System Through Diverse Stimuli Because They Are Trying to Perform Functions That Are no Longer Present in Our Species

 This concept is part of an “atavist” hypothesis of pemphigus vulgaris based on the mechanism of shedding in reptiles [75, 76].

### 3.2. Classes I and II MHC

In vertebrates, allorecognition depends on proteins encoded by MHC genes. An MHC-like region is certainly very ancient and is believed to be present in the common ancestor of proto- and deuterostomes [77]. The function of MHC is to present antigens to T cell receptors. It has been proposed that the MHC region arose as a result of chromosomal duplications. In higher vertebrates, MHC is represented by two distinct classes, MHC I and MHC II. In the intracellular processes, the participation of chaperons and transporter molecules is necessary. The most likely hypothesis is that the ancestral MHC molecule had a class II-like structure which later gave rise to a class I molecule [78, 79].

#### 3.2.1. Class I MHC

This is widely distributed and is expressed on most nucleated cells. As a general rule, antigens generated within the cell (endogens) are processed in the cytosolic pathway, transported with class I MHC molecules to the plasma membrane of most nucleated cells and presented to T-cells. Class I MHC requires proteasomes for antigen generation. These structures are phylogenetically ancient as they are found both in bacteria and eukaryotes. Peptides generated by the proteasome in *Drosophila *and yeast, which lack MHC, are rerouted towards a new biochemical pathway *via *the peptide transporters. Agnathans lack the ability to produce immunoproteasomes [80].

#### 3.2.2. Class II MHC

 Antigens taken up by phagocytosis (exogenous) are processed in the endocytic pathway, transported with class II MHC molecules, and presented in the membrane of macrophages, dendritic cells, and B cells. They are assembled within the rough endoplasmic reticulum where they associate with a glycoprotein called invariant chain (Ii, CD74) which prevents premature binding of any endogenously derived peptides. Afterwards, CD74 is digested by cathepsins S and L in order to free the binding fragment (CLIP). The removal of CLIP and peptide loading requires HLA-DM, an endosome-resident accessory molecule. In mammalian B cells, peptide loading is further modulated by another molecule, HLA-DO. The invariant chain glycoprotein, CD74, is found only in the gnathostome vertebrates. Several cathepsins seem to have been associated with class II MHC for peptide presentation several times during evolution at the level of exogenous peptide processing and processing of CD74 [81]. Class II MHC polymorphisms have been studied and their presence is a risk factor for various autoimmune diseases, for example, HLA DRB1*04 in RA, HLA DRB1*0301 in SLE o HLA DR1*0301-DQB1*0201 in SS [82]. Sometimes the risk is caused by the combination of polymorphisms (haplotypes) such as the 8.1 ancestral haplotype [83]. The presence of these molecules is associated with the common genesis of several autoimmune diseases (autoimmune tautology) [84]. In other mammals, similar associations have been shown [85].

## 4. Influence of the Third Form of Evolutionary Memory in the Autoimmune Phenomena

Memory related to neuronal function in relation to autoimmune phenomena is a crucial factor for storing diverse experiences during life.

### 4.1. Neuroimmunoendocrine Network

An emotion such as stress triggers endocrine responses which, in turn, affect the immune system and cause its activation and inappropriate response in the setting of autoimmune and infectious diseases. Many retrospective studies found that a high number of patients reported uncommon emotional stress before disease onset. Other studies suggest that stress is not only a participating factor but may also be a cause of disease exacerbations. Unfortunately, it is a vicious cycle because stress not only causes disease but the disease itself causes considerable stress in patients [86]. Neuroendocrine hormones triggered during stress may lead to immune dysregulation or altered or amplified cytokine production thus resulting in autoimmune diseases. Various types of transmitter substances in the neuroendocrine-immune network include epinephrine, norepinephrine, acetylcholine, substance P, vasoactive intestinal peptide, glucagon, insulin, cytokines, and growth factors. The stress response and induction of a disregulation in the cytokine balance can trigger the hypothalamic-pituitary-adrenal axis and sympathetic nervous system [87].

### 4.2. Neuron-Glial Relationship

Another type of information stored in the brain is related to the experience of pain. Somatic pain induces an immediate response that generates the reflex which moves the injured body part and prevents an increase in the damage. A second form of pain is the subacute or chronic somatic pain that is caused by the rest of the affected area during the repair process. Another form of pain is the neuropathic pain which is due to the persistent response that is generated after an injury to peripheral nerve structures [88]. In this condition the microglia, which are the resident macrophages of the brain and spinal cord, plays an important role. It seems that the nerve injury activates the receptor TLR4 which is only expressed in the central nervous system on microglia. Genetically altered mice lacking TLR4 showed markedly reduced microglial activation after nerve injury as well as reduced sensitivity to pain [89]. Another candidate trigger for microglial activation after nerve injury is fractalkine, a CX3C chemokine, expressed on the surface of neurons [90]. But, how does microglia excite sensory neurons? Many researchers have suggested that proinflammatory cytokines secreted by activated microglia increase neural excitability and sensitivity to pain when injected into the spinal cords of rats. It is possible that many brain functions allocated exclusively to neurons are dependent on a neuron-microglia interaction. Ontogenetically both microglia and other glial cells are important in the process of migration and location of neurons in the central nervous system and are also closely related to their control electrolyte and nutrient supply [91]. Not only glial cells but also neurons respond to the presence of several cytokines [92]. The concept of integrated neuroendocrine-immune regulation has now been widely accepted although the physiological impact on normal development and homeostasis or conditions of mental stress or physical disease are still poorly understood [93]. The actions of cytokines via their receptors may also have physiopathological implications in other conditions such as multiple sclerosis, Alzheimer's disease, and AIDS dementia syndrome [94, 95].

### 4.3. Role of IL-1 and IL-1Rs

 A well-known example is the role of IL-1 in the stress-axis, the hypothalamis-pituitary-adrenal (HPA) axis in mammals [96]. This axis is activated by inflammatory cytokines like IL-1 through the release of the corticotrophin releasing hormone (CRH) and proopiomelanocortin (POMC)-derived adrenocorticotropic hormone (ACTH) from the hypothalamus and anterior pituitary gland, respectively [97, 98]. Upon the release of ACTH, corticosteroid production, and release from the adrenal cortex will be stimulated which, in turn, causes redistribution of leukocytes, inhibition of antibody production and release of inflammatory cytokines. IL-1R family members are encoded on the X chromosome and they are present in multiple teleost orthologues as well as in chickens. In mammals, they are abundantly expressed in the hippocampal memory system of the brain and therefore might contribute to brain development or function [99]. Interestingly, mutations in this loci are responsible for a hereditary form of mental retardation [100, 101].

## 5. Influence of the Fourth Form of Evolutionary Memory in the Autoimmune Phenomena

The fourth form of mankind's evolutionary memory is culture, and with this, there are many conditions that are related to the genesis of autoimmune phenomena. Humans who have changed to an urban environment have undergone changes in their exterior stimuli which their biological structure cannot be adequately adapted to given the limited period for that adaptation.

### 5.1. Changes in the Diet

Dietary modifications for humans that led to protein-calorie malnutrition or otherwise to weight excess have been assessed and taken into account when seeking nutritional factors associated with development of autoimmunity [102, 103]. The lack of exposure to various antigens in early childhood, including the newly born and those growing up in an “aseptic,” environment results in a very low exposure to antigens required for the development of cell-mediated immunity (leading to better and safer protection) and, at later stages, requires an antibody-mediated protection which can lead to allergy and autoimmunity phenomena [104].

### 5.2. Changes in Exposure to Ultraviolet (UV) Radiation

 It is well known that industrialization decreases exposure to sunlight with the consequent development of vitamin D deficiency, now a well-studied factor related to autoimmunity [105]. At the other extreme, the overexposure to sunlight due to working conditions or the cultural practice of changing the color of the skin (tanning) increases UV radiation. This is related to the development or exacerbation of cutaneous lupus and SLE by different mechanisms [106, 107].

### 5.3. Effects Related to Jobs and Habits

 Some jobs are related to risks of SLE. For example, school teachers are exposed to many viruses that can be the basis for an abnormal immune response in a genetically predisposed individual [108]. Various habits such as smoking [109] or exposure to smog-related particles [110] are also associated with a similar outcome.

### 5.4. Effects of Exposure to Drugs

 With the evolution of human culture, pharmacology also developed and diverse drugs could induce autoimmune phenomena through various factors including epigenetic ones (Example: procainamide, hydralazine, Chlorpromazine, isoniazid, phenytoin, and penicillamine) [111]. Women have a greater predisposition to developing autoimmunity in part as a result of the effect of estrogen, which, when used for therapeutic or contraceptive reasons, increases the risk [112].

### 5.5. Effects of Migrations and Social Aspects

 It has been postulated that older factors such as migration and exposure for long periods of time to different forms of environment were determining factors for the development of different human races which have different risks for the development of autoimmune diseases [113–116]. More recently there has been a genetic mixing of races due to migration and risk conditions for developing diseases have changed for each race. Race can also be modified without requiring migration; for instance, by religious or political influences that encourage the choice of partner for reproduction in order to ensure a race with certain characteristics which are more related to beauty and purity [117]. Human society has the same dynamic processes as nature, which has a tendency to maintain a status of “entropy” within constant “chaos” in physicochemical terms, or constant “changes” in socioanthropological terms [118].

## 6. Conclusions

One way to understand autoimmunity is through knowledge of the biological significance of evolution. Since a specialized system for defense against microorganisms was set up, living things have theoretically been vulnerable to developing autoimmune phenomena. The existence of different regulatory mechanisms can only be explained as strategies that were developed to avoid these phenomena of self-destruction. The human species have a cumulative evolution of the most complex mechanisms of innate and acquired immunity which makes it very vulnerable to autoimmunity especially when the regulatory mechanisms fail. Other risk factors are not inherited and are the product of the evolution of the brain and culture in the human species.
